# Effects of vitamin D deficiency on the improvement of metabolic disorders in obese mice after vertical sleeve gastrectomy

**DOI:** 10.1038/s41598-021-85531-9

**Published:** 2021-03-16

**Authors:** Jie Zhang, Min Feng, Lisha Pan, Feng Wang, Pengfei Wu, Yang You, Meiyun Hua, Tianci Zhang, Zheng Wang, Liang Zong, Yuanping Han, Wenxian Guan

**Affiliations:** 1 Department of General Surgery, Nanjing Drum Tower Hospital Clinical College of Nanjing Medical University, Nanjing, China; 2 Department of General Surgery, The Affiliated Hospital of Yangzhou University, Yangzhou, China; 3 Department of General Surgery, Nanjing Drum Tower Hospital, The Affiliated Hospital of Nanjing University Medical School, No. 321 Zhongshan Road, Nanjing, 210008 China; 4 Center for Growth, Metabolism and Aging, Analytical and Testing Center, Key Laboratory of Bio-Resource and Eco-Environment, College of Life Sciences, Sichuan University, No. 24 South Section 1, Yihuan Road, Chengdu, 610065 China; 5 Department of Pathology, The First Affiliated Hospital of Yangzhou University, Yangzhou, China; 6 Department of Gastrointestinal Surgery, Changzhi People’s Hospital, The Affiliated Hospital of Changzhi Medical College, Changzhi, Shanxi China

**Keywords:** Fat metabolism, Cystic fibrosis

## Abstract

Vertical sleeve gastrectomy (VSG) is one of the most commonly performed clinical bariatric surgeries for the remission of obesity and diabetes. Its effects include weight loss, improved insulin resistance, and the improvement of hepatic steatosis. Epidemiologic studies demonstrated that vitamin D deficiency (VDD) is associated with many diseases, including obesity. To explore the role of vitamin D in metabolic disorders for patients with obesity after VSG. We established a murine model of diet-induced obesity + VDD, and we performed VSGs to investigate VDD's effects on the improvement of metabolic disorders present in post-VSG obese mice. We observed that in HFD mice, the concentration of VitD3 is four fold of HFD + VDD one. In the post-VSG obese mice, VDD attenuated the improvements of hepatic steatosis, insulin resistance, intestinal inflammation and permeability, the maintenance of weight loss, the reduction of fat loss, and the restoration of intestinal flora that were weakened. Our results suggest that in post-VSG obese mice, maintaining a normal level of vitamin D plays an important role in maintaining the improvement of metabolic disorders.

## Introduction

Obesity and its related comorbidities affect 2.1 billion people worldwide^1^. In last decade, bariatric surgery has emerged as an effective treatment for obese individuals because of its sustained ability to reduce body weight^2–4^. Till now, Roux-en-Y gastric bypass (RYGB) and vertical sleeve gastrectomy (VSG) are commonly conducted bariatric surgeries. These two surgical approaches can lead to significant weight loss and improve glucose tolerance in both rodent models and humans^5,6^. Recently, VSG has gradually become more popular due to its simplicity, fewer complications and lower mortality^7^.

Vitamin D is the precursor of the active steroid hormone 1,25-dihydroxyvitamin D_3_ (1,25(OH)_2_D_3_). Vitamin D has been investigated for its effects on the regulation of intestinal flora^8,9^ and toward increasing tumor immunity^10–12^. In humans, vitamin D is synthesized by skin exposure to ultraviolet light (mainly UVB, 280–320 nm) and by its direct ingestion in fortified foods or supplements^13,14^. Once in the body, vitamin D is hydroxylated in the liver by cytochrome P450 enzyme (CYP2R1) to the intermediate metabolite, 25-hydroxyvitamin D3 (25(OH)D_3_), and then the circulating 25(OH)D_3_ is converted to the active form of vitamin D-1,25-dihydroxyvitamin D3 (1,25(OH)_2_D_3_) by CYP27B1 in the kidneys, which is the canonical pathway^15,16^. In addition, novel CYP11A-mediated pathways have been identified^17^. Slominski AT found that CYP11A1 hydroxylated the side chains of D3 and D2 in turn, and the product can be further hydroxylated by CYP27B1, CYP27A1 and CYP24A1. This is an alternative to the classical pathway, but its physiological importance remain to be confirmed^17^. Another study by Slominski AT reported a previously unrecognized in vivo pathway of vitamin D3 (D3) metabolism to generating novel D3-hydroxy derivatives different from 25-hydroxyvitamin D3 [25(OH)D3] and 1,25(OH)2D3^18^. These new derivatives were produced by placenta, adrenal glands and epidermal keratinocytes, including 20-hydroxyvitamin D3 [20(OH)D3], 22(OH)D3, 20,23(OH)2D3, 20,22(OH) 2D3, 1,20(OH)2D3, 1,20,23(OH)3D3, 17,20,23(OH)3D3. It provides evidence for the new pathway of CYP11A1 to initiate D3 metabolism in vivo, and its products show organ/cell type specificity and are modified by CYP27B1 activity. These findings define intermediates as natural products/endogenous biological regulators, which is different from the current dogma that vitamin D is only activated by the sequence D3 → 25(OH)D3 → 1,25(OH)2D3^18^.

Circulating 25(OH)D_3_ and 1,25(OH)_2_D_3_ both bind to vitamin D in the blood, and are inactivated via 24-hydroxylation by CYP24A1 and ultimately catabolized^19^. Circulating 25(OH)D_3_ is the predominant form of vitamin D. The Institute of Medicine (IOM) defines vitamin D deficiency (VDD) as a serum 25(OH)D_3_ level ≤ 20 ng/mL, whereas the Endocrine Society defines VDD as ≤ 30 ng/mL^20,21^. Currently, due to air pollution, insufficient sunlight exposure, and altered dietary composition, vitamin D deficiency become more common, and it is associated with many diseases including asthma, heart disease, depression, colorectal cancer, and obesity^22–29^.

The mechanisms underlying the effects of bariatric surgery are not yet clear. It has been proposed that potential mechanisms of bariatric surgery may be involved in the body's carbohydrate metabolism, including gut hormones, hypocaloric restriction, intestinal flora metabolism^30–32^, bile acid physiology^33–35^, and a reduction in the level of lipopolysaccharides (LPS) after surgery^36,37^. Our previous research showed that a high-fat + vitamin D-deficient diet hampers the enterohepatic circulation of bile acids, leading to non-alcoholic steatohepatitis (NASH)^38^, and we reported that this diet aggravated the impairment of intestinal function and affected nutrient absorption in mice with metabolic syndrome^8^. In the present study, we established a murine model of diet-induced obesity with VDD, and we performed VSG surgery to investigate whether VDD affects the improvement of metabolic disorders after VSG.

## Research design and methods

### Animals and treatment

All of the animal experiments complied with the U.S. National Institutes of Health Guidelines for the Care and Use of Laboratory Animals and complied with the ARRIVE guidelines, the procedures were approved by The Institutional Animal Care and Use Committee of Sichuan University. We purchased 4-week-old male C57BL/6J mice from Beijing HFK Bioscience. The mice were housed under SPF conditions at the animal center at the College of Life Sciences, Sichuan University, and they were given an acclimatization period of 1 week upon their arrival at the animal center. The mice were maintained in a controlled environment (12:12 h light–dark cycle) with free access to food and water.

The diet-induced obesity (DIO) model was established as described^39^. Each mouse was fed one of four types of diet for 14 weeks: (1) control chow with VD_3_ at 1000 IU/kg (standard AIN93 formula), comprising the control (Ctrl) group (n = 6); (2) vitamin D-depleted control chow, as the VDD group (n = 6); (3) a high-fat diet (60% calories from fat) with a VD_3_ supplement at 1000 IU/kg, as the high-fat diet (HFD) group (n = 12); or (4) high-fat chow without the vitamin D supplement, comprising the HFD + VDD group (n = 12). The compositions of the four types of diet and the vitamin kit are listed in Tables 1 and 2, respectively.

**Table 1:** Composition of four types of diet used in the study.

Ingredient	Control (g/kg)	VDD (g/kg)	HFD (g/kg)	HFD + VDD (g/kg)
Amino acids	195.6	195.6	238.8	238.8
Cornstarch	397.5	397.5	0	0
Dextrinized cornstarch	132	132	179.7	179.7
Sucrose	100	100	92.6	92.6
Fiber	50	50	66.3	66.3
Soybean oil (no additives)	70	70	33.1	33.1
Lard	0	0	319.7	319.7
Mineral mix	35	35	46.4	46.4
Vitamin mix	10 (VD_3_ 1000 IU/kg)	10 (− VD_3_)	10 (VD_3_ 1000 IU/kg)	10 (− VD_3_)
NaHCO_3_	7.4	7.4	9.8	9.8
Choline bitartrate (41.1% choline)	2.5	2.5	3.3	3.3
Tert-butylhydroquinon	0.1	0.1	0.1	0.1
Total	1000	1000	1000	1000

**Table 2:** Composition of vitamin kit.

Vitamin	mg or U/kg diet
Nicotinic acid, mg	30
Pantothenate, mg	15
Pyridoxine, mg	6
Thiamin, mg	5
Riboflavin, mg	6
Folic acid, mg	2
Vitamin K, mg	750
D-Biotin, mg	200
Vitamin B-12, mg	25
Vitamin A, IU	4000
Vitamin D_3_, IU	1000
Vitamin E, IU	75

After the 14-week diet period, we randomly subdivided the HFD mice and the HFD + VDD mice into two body weight-matched groups (VSG and sham) and perform the VSG surgery or a sham surgery for each mouse as described below. After the recovery during the immediate postoperative period, each mouse was then fed its presurgery diet for 10 weeks. The food intake and body weight of each mouse were recorded weekly.

### VSG and sham surgeries

The VSG surgery was performed with the mouse under general anesthesia. Inhalational anesthesia was induced and maintained using isoflurane and oxygen. The lateral 80% of the stomach was resected, leaving a tubular gastric remnant in continuity with the esophagus superiorly and the pylorus and duodenum inferiorly as described^33^. For the sham procedure, the mouse underwent the same surgical protocol except that the abdominal cavity was simply held open for approx. 30 min (the estimated duration of the gastrectomy) so that surgical stress that was similar to that of the VSG group was experienced by the mouse.

### Glucose tolerance tests (GTTs), insulin tolerance test (ITTs), and HOMA-IR analysis

Mice were fasted for 6 h or 4 h before undergoing a glucose tolerance test (GTT) or insulin tolerance test (ITT), respectively^8^. For each GTT or ITT, glucose (1 g/kg body weight, 20% glucose solution) or insulin (1 U/kg body weight) was injected intraperitoneally. Blood glucose was tested by a glucose meter (Accu-Chek Active, Roche, Mannheim, Germany) with 5 μL of blood that had been collected from the tip of the tail vein. The plasma insulin concentrations were measured by an enzyme-linked immunosorbent assay (ELISA) kit (DRE31141, RB).

For the analysis of homeostatic model assessment of insulin resistance (HOMA-IR) values, the following formula was used: (glucose concentration × insulin concentration)/22.5.

### Plasma biochemistry

The plasma LPS concentration was determined by a limulus amebocyte extract kit (CE80545S, Xiamen Bioendo Technology, Xiamen, Fujian, China) The plasma LPS content was analyzed following the manufacturer's instructions. The levels of blood glucose, serum triglycerides, alanine aminotransferase (ALT), and aspartate aminotransferase (AST) were measured by automated instruments at the Chengdu Public Health Clinical Medical Center (Chengdu, China).

### Histological analysis of adipose tissues

Adipose tissues were fixed in 4% paraformaldehyde and then embedded in paraffin. Oil red O staining and hematoxylin and eosin (H&E) staining were performed as described.

### RT-qPCR analysis

We extracted RNA according to the previous practices^8^. Firstly, total RNA was isolated using Trizol (Transgen, Beijing, China), and transcribed into cDNA using the PrimeScriptTM RT reagent Kit (Cat. RR047A, TaKaRa, Shiga, Japan). The RT-qPCR was performed using a qPCR instrument (#cfx96, Bio-Rad, Hercules, CA). The primer sequences are listed in Table 3. All the expression levels of genes were normalized by GAPDH.

**Table 3:** Primers used for RT-qPCR analysis.

Mouse gene	Forward 5′ → 3′	Reverse 5′ → 3′
Claudin-2	CCTTCGGGACTTCTACTCGC	TCACACATACCCAGTCAGGC
Occludin	ATGTCCGGCCGATGCTCTC	TTTGGCTGCTCTTGGGTCTGTAT
ZO-1	ACCCGAAACTGATGCTGTGGATAG	AAATGGCCGGGCAGAACTTGTGTA

### Quantification of short-chain fatty acids

Short-chain fatty acids (SCFAs) were extracted from the cecal contents of the mice as described^40^. One and half gram of cecum contents was added to 5 mL of water, and the mixture homogenized for 3 min. The pH of the cecal suspension was adjusted to 2.0–3.0 by adding 5 M HCl solution. This mixture was then transferred to polypropylene tubes, and centrifuged for 30 min at 3000 rpm. Finally, the supernatant was filtered with a 0.45-μm microfilter so that we can inject it into a gas chromatograph for the quantification of SCFAs.

### Gut microbiota analysis

For the analysis of the gut microbiota of the mice, the sequencing library preparation, sequencing, and data analysis were performed by Novogene (Beijing, China). Detailed information on the microbiome analysis can be found in the Supplementary Information.

### Statistical analyses

The data recording, processing, and calculation were completed using Microsoft Excel 2010 and GraphPad Prism 5.0. The data are presented as mean ± standard error of the mean (SEM). Comparisons across groups were assessed with Student's t-test or a one-way analysis of variance (ANOVA) followed by Tukey multiple comparison testing. Statistical significance was accepted at p < 0.05.

### Ethics approval and consent to participate

This study was approved by the institutional review board of Sichuan University and Nanjing Drum Tower Hospital Clinical College of Nanjing Medical University. All methods were carried out in accordance with relevant guidelines and regulations in manuscript.

## Results

### VDD attenuated the improvement of hepatic steatosis, the maintenance of weight loss, and the reduction of fat loss following VSG in obese mice

To determine whether VDD affects the changes in related physiological effects following bariatric surgery, we performed the VSG surgery on mice with diet-induced obesity (DIO). After the 14 weeks of the high-fat diet, the body weights of the DIO mice (i.e., both the HFD mice and the HFD + VDD mice) were significantly increased compared to those of the mice fed a normal diet as the high calories and fat (Supplementary Fig. S1A,B), this result shows HFD could successfully induced diet-induced obesity (DIO) in mice compared to normal diet; and the plasma VD_3_ concentrations of the HFD + VDD mice were significantly lower than those of the HFD mice (p < 0.01) (Supplementary Fig. S1C,D). The blood glucose levels of the DIO mice were also increased (Supplementary Fig. S1E).

**Figure 1:**
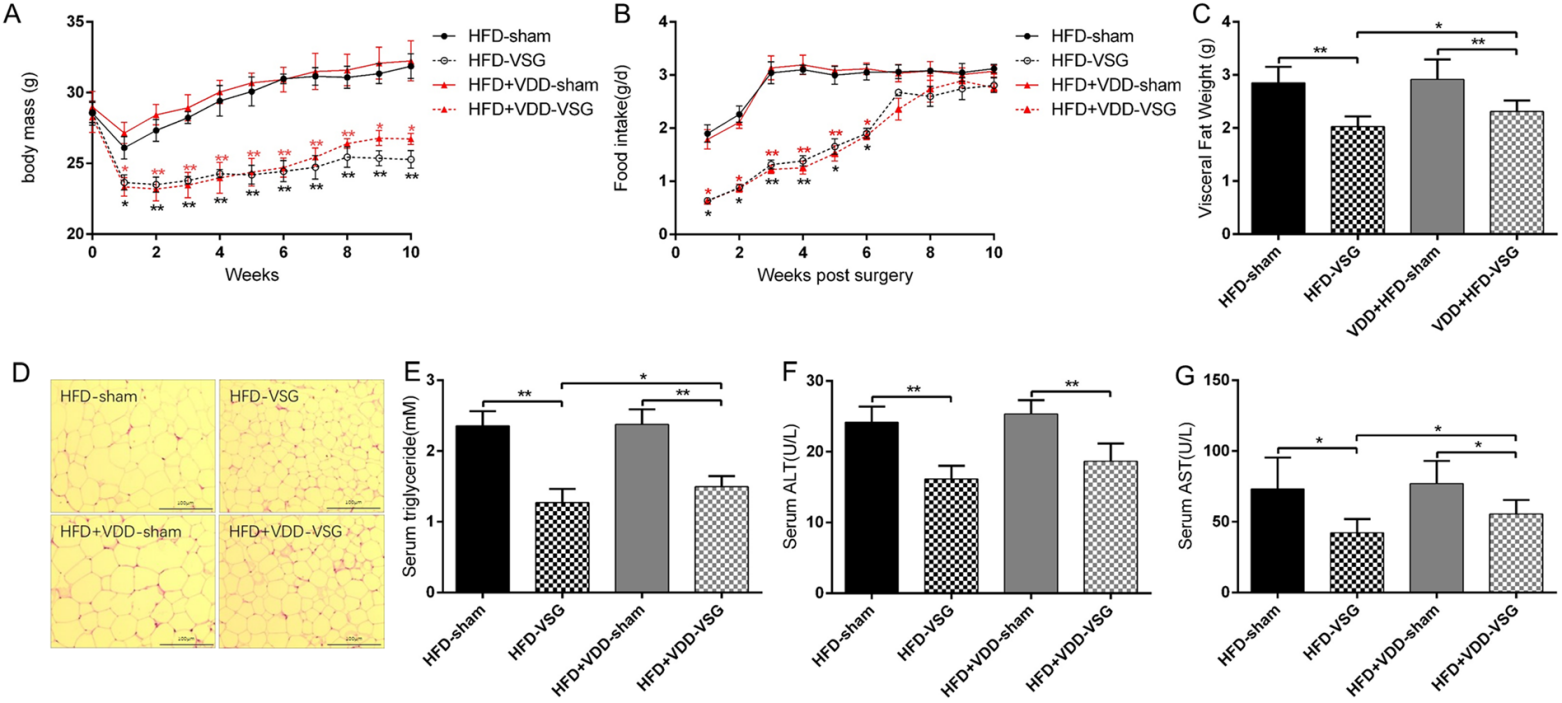
VDD weaken the improvement of hepatic steatosis, maintenance of weight loss and reduction of fat loss following VSG in obese mice. Mice were fed with HFD and HFD + VDD to induce DIO, and then subjected to VSG or sham surgical procedures. (**A**) Body weight of mice after surgery. (**B**) Food intake of mice after surgery. (**C**) Visceral fat weight of mice after surgery. (**D**) H&E staining of adipose tissue sections. (**E**–**G**) Serum triglyceride levels, Plasma alanine aminotransferase (ALT) and aspartate aminotransferase (AST) levels. *p < 0.05, **p < 0.01 versus sham-operated mice by one-way ANOVA with Dunn’s post-test. Values are presented as the mean ± SEM (n = 6 per group). Scale bars, 100 μM.

We next performed VSG or sham surgery in both HFD mice and HFD + VDD mice. In the first 6 weeks after the surgery, the HFD mice and HFD + VDD mice displayed similar losses in body weight compared to their respective sham-operated mice (Fig. 1A). Over the course of the first 6 weeks, decreased food intake was observed in the mice that had undergone VSG surgery compared to the sham-operated mice (Fig. 1B). By week 7, the food intakes among all mice were similar. In addition, the body weights of both the HFD-VSG mice and the HFD + VDD-VSG mice remained lower than those observed in the sham-operated mice, despite the lack of a significant difference in all of the groups' food intake after week 7. These data are consistent with those of previous studies^33,41^. The HFD-VSG mice and the HFD + VDD-VSG mice maintained their weight loss throughout the post-VSG period (Fig. 1A), but the body weights of the HFD-VSG mice remained lower than the weights of the HFD + VDD-VSG mice after week 8 (Fig. 1A).

Our measurements of the visceral fat weights of the HFD-VSG mice and HFD + VDD-VSG mice showed that compared with the corresponding groups of sham mice, the visceral fat weights of the VSG mice fell dramatically post-VSG (p < 0.01, each), and there was a significant difference in the visceral fat weights between HFD-VSG mice and HFD + VDD-VSG mice (p < 0.05) (Fig. 1C).

By conducting the histological analysis of adipose tissue, we observed that the VSG surgery significantly reduced the adipocyte hypertrophy steatosis in the mice fed the HFD compared to the sham mice, but this change was not clear in the HFD + VDD mice (Fig. 1D). By conducting the hematological testing, we observed decreased serum triglyceride levels (Fig. 1E) and decreased serum ALT and AST levels after VSG. In addition, compared to the values of the HFD + VDD-VSG mice, the ALT and AST levels among the HFD-VSG mice were lower (Fig. 1F,G). These results demonstrate that VDD weakened the role of the VSG surgery in reducing hepatic steatosis in mice, maintaining their weight loss, and reducing their fat mass, which indicated that maintaining a normal level of vitamin D is important in obese mice after VSG surgery.

### VDD affected the post-VSG glucose control

**Figure 2:**
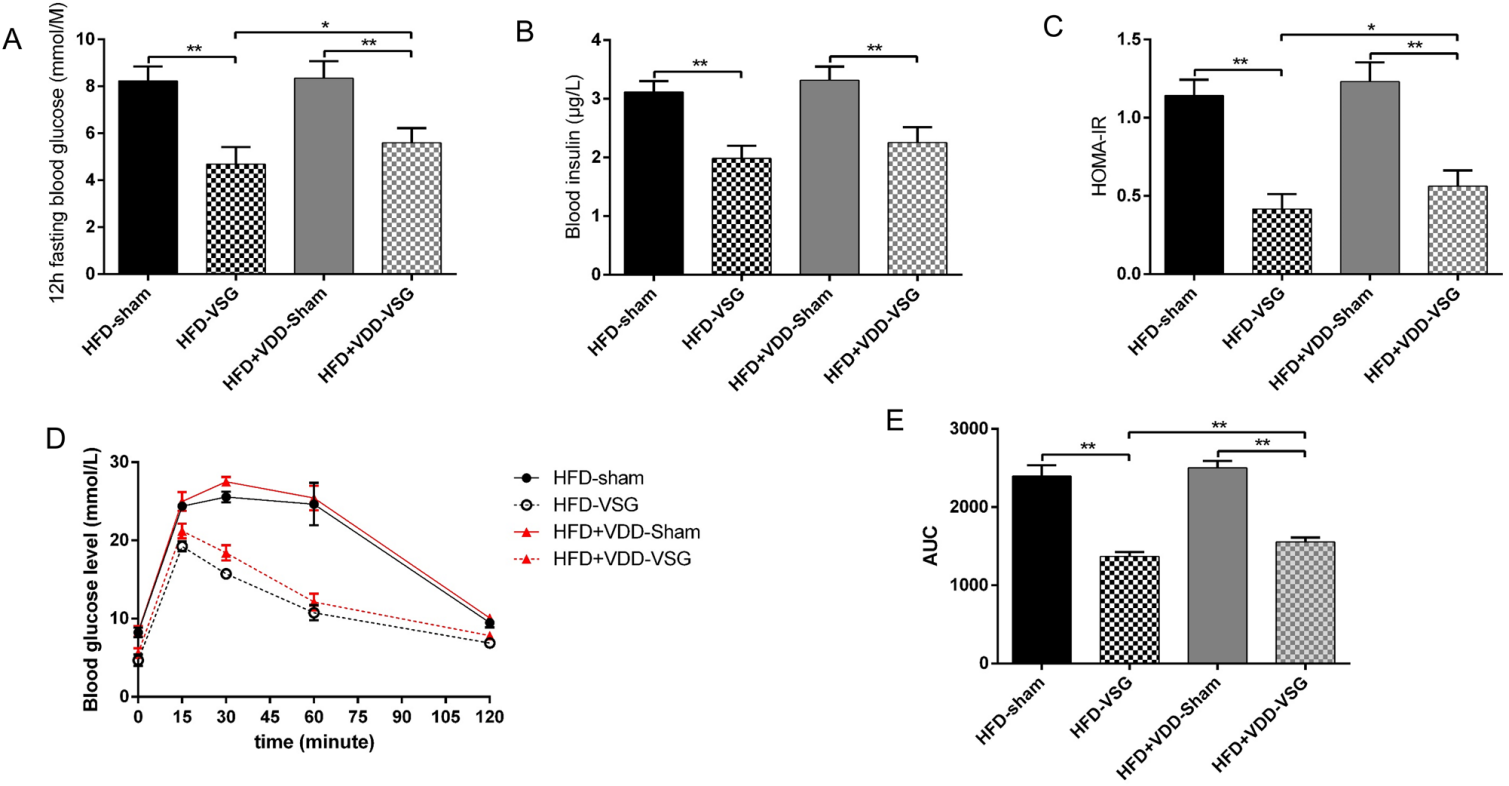
VDD affect the improvement in glucose tolerance and insulin sensitivity after VSG. (**A**) Fasting blood glucose level of mice. (**B**) Fasting blood insulin level of mice. (**C**) HOMA-IR was measured in mice at 12 weeks after surgery. (**D**,**E**) Intraperitoneal glucose tolerance test and area under the curve from 0 to 120 min in HFD and HFD + VDD mice after intraperitoneal injection of a bolus of 1 g/kg d-glucose. *p < 0.05, **p < 0.01 versus sham-operated mice by way of one-way ANOVA with Dunn’s post-test. Values are presented as the mean ± SEM (n = 6 per group).

Previous study showed that baseline glucose was significantly higher in mice which were fed with high-fat diet for 1 week, and a stable hyperglycemia and insulin resistance appeared over time^42^. However, VSG surgery could provides an substantial improvement of glucose^43^. Therefore, in our study, to investigate whether VDD affects the glucose homeostasis after VSG surgery, we analyzed the results of the glucose tolerance tests and the metabolite values in all of the mice that had undergone the VSG. Prior to the VSG surgery, no significant difference in fasting blood glucose levels or in glucose tolerance was observed between the obese HFD and HFD + VDD mice (Supplementary Fig. S2A,B), but the fasting blood glucose and insulin levels measured postoperatively were lower in the HFD-VSG mice compared to those in the HFD-sham controls; moreover, the fasting blood glucose and insulin levels of the HFD + VDD-VSG mice were also lower than those of the HFD + VDD-sham controls (Fig. 2A,B). The fasting blood glucose levels of the HFD-VSG mice were significantly lower than those of the HFD + VDD-VSG mice (p = 0.0403). The insulin levels of the HFD-VSG mice were also lower than those of the HFD + VDD-VSG mice, but the difference was not significant (p = 0.0849) (Fig. 2A,B).

In line with these findings, the results of the HOMA-IR analysis demonstrated that the insulin resistance of the HFD-VSG mice and HFD + VDD-VSG mice were diminished compared to that of the HFD-sham and HFD + VDD-sham mice respectively. The insulin resistance values also differed between the HFD-VSG and HFD + VDD-VSG mice (Fig. 2C). In the intraperitoneal GTT, the glucose tolerance was improved in the HFD-VSG mice challenged after surgery compared to the HFD-sham mice (Fig. 2D), and the values of the HFD + VDD-VSG and HFD + VDD-sham mice also differed (Fig. 2D). Notably, the HFD-VSG mice exhibited an improved ability to clear glucose, an observation reflected as a 43% reduction in the area under the curve (AUC) relative to the values of the HFD-sham mice. However, compared to the HFD + VDD-sham mice, there was only a 38% reduction in the AUC of the HFD + VDD-VSG mice (Fig. 2E). These results shown that VDD changed the VSG's effect of improving the glucose tolerance of the mice, which demonstrate a normal level of vitamin D is crucial for the regulation of glucose in post-VSG obese mice.

### VDD weakened the effects of VSG on intestinal inflammation and permeability improvement in obese mice

**Figure 3:**
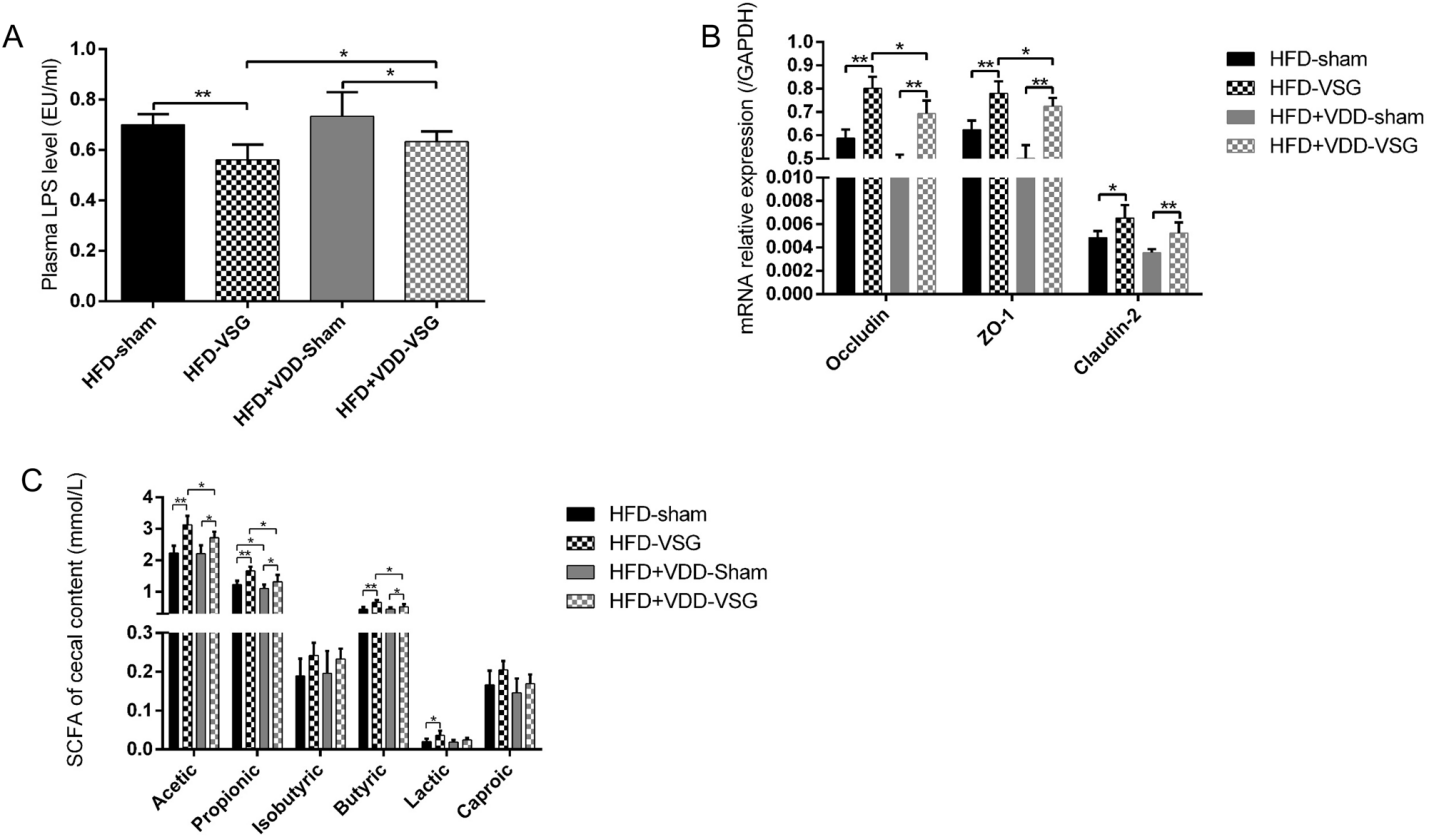
VDD weaken the effect of VSG on intestinal inflammation and permeability improvement in obese mice. (**A**) Plasma LPS level after surgery. (**B**) Expression of Occludin, ZO-1, and claudin-2 in ileum region was determined by RT-qPCR analysis. (**C**) Concentration of SCFA (mmol/L) (acetic acid, propionic acid, isobutyl acid, butyric acid, lactate acid and caproic acid) of mice after surgery. *p < 0.05, **p < 0.01 versus sham-operated mice by way of one-way ANOVA with Dunn’s post-test. Values are presented as the mean ± SEM (n = 6 per group).

To investigate the effect of VDD on small intestinal inflammation and permeability after VSG, we measured the plasma LPS of the mice after VSG. The plasma LPS levels were significantly decreased after the VSG surgery; in addition, compared to the values of the HFD + VDD-VSG mice, the LPS levels of the HFD-VSG mice were lower (Fig. 3A).

Since tight junctions (TJs) maintain the enteral epithelial barrier and defects in TJs may result in gut impairment, we analyzed the changes of key components of the TJs expressed in the ileum. Compared to the expressions in the HFD-sham and HFD + VDD-sham mice, the mRNA relative expressions of occludin, ZO-1, and claudin-2 in the ileum regions of the HFD-VSG and HFD + VDD-VSG mice were increased. The mRNA relative expressions of occludin and ZO-1 in the ileum regions of the HFD + VDD-VSG mice were significantly decreased compared to those of the HFD-VSG mice (p < 0.05), but there was no difference in claudin-2 expression between the HFD-VSG and HFD + VDD-VSG mice (p = 0.0652) (Fig. 3B).

Taking these results into account along with the changes of plasma LPS, we speculate that the VSG postoperative intestinal permeability change is associated with the change of LPS levels, and the increased expression of TJs is associated with a decreased LPS level. We also suspect that after a VSG, VDD suppresses the increased expression of TJs and leads to the elevation of LPS, ultimately affecting the integrity of the ileal mucosal lining.

We next measured the changes of SCFAs of the cecal contents of mice after they had undergone a VSG. The changes of SCFA in cecal contents are illustrated in Fig. 3C. Compared to the contents in the sham mice, the average SCFA content in the cecal contents of the VSG mice was higher, among which acetic acid, propionic acid, and butyric acid were more readily observed; the other SCFA including isobutyric acid, lactic acid, and caproic acid also showed some between-group differences, but these were not significant. Regarding acetic acid, the HFD-VSG mice and HFD + VDD-VSG mice showed significantly higher mean values compare to the respective HFD-sham and HFD + VDD-sham groups. In addition, the acetic acid values of the HFD-VSG mice differed from those of the HFD + VDD-VSG mice. The same type of findings were observed for propionic acid and butyric acid. The results illustrated that a normal level of vitamin D had effects on intestinal inflammation and permeability in post-VSG obese mice.

### VDD affected the alleviation of post-VSG gut dysbiosis

**Figure 4:**
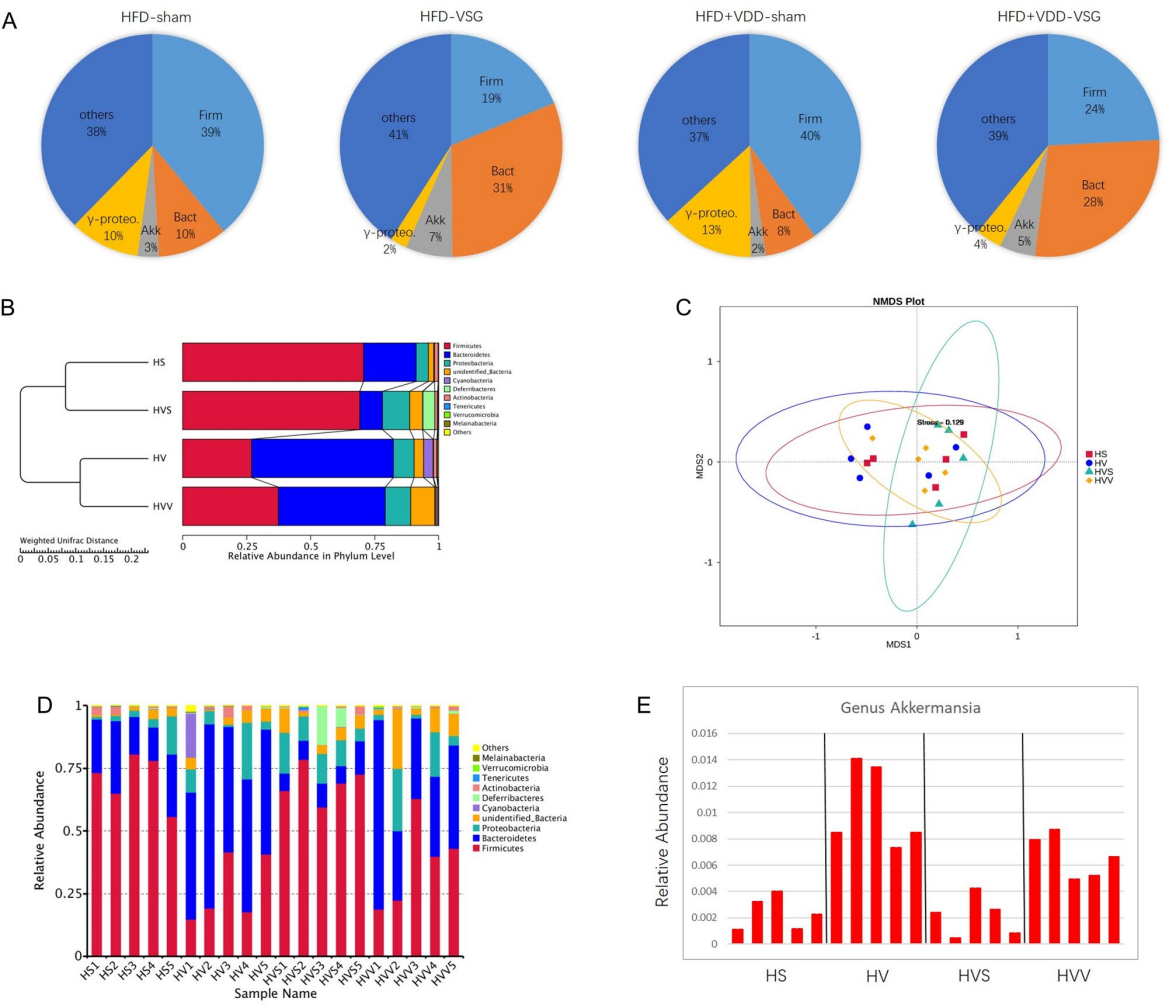
VDD affect the alleviation of gut dysbiosis after VSG. The microbiota in the ileum and feces of the mice described in Fig. 1 was examined. (**A**) The abundances of phyla Firmicutes (Firm) and Bacteroidetes (Bact), Akkermansia muciniphila (Akk, spp.), genus g-Proteobacteria in the feces were detected by 16S rDNA-qPCR (n = 6 for each group). (**B**) The bacteria population in the lumen of ileum was determined by high-throughput sequencing analysis (n = 5 for each group). UPGMA (unweighted pair-group method with arithmetic mean) clustering based on weighted unifrac distance of the top 10 relative abundant phyla. (**C**) NMDS (non-metric multidimensional scaling) plot, every point showed one mouse, different colors signified different groups, and the stress = 0.129. (**D**) The relative abundance of bacteria at phylum levels in the ileal lumen. (**E**) The relative abundance of Akkermansia in the ileal content.

The elevation of the plasma LPS levels by the high-fat feeding may have been the result of gut dysbiosis and impairment of the intestinal interface. We observed that at the phylum level, Firmicutes was dominant over Bacteroidetes in the HFD-sham mice (Fig. 4A). This result was in agreement with clinical findings of increased Firmicutes and decreased Bacteroidetes in obese patients^44,45^. In contrast, in the HFD-VSG mice the abundance of Firmicutes was reduced and that of Bacteroidetes was greatly increased. These observations indicated that the VSG surgery can restore eubiosis, reducing Firmicutes and restoring Bacteroidetes, a major group of Gram-negative microbes that generate endotoxin at their death.

In the group of HFD + VDD-sham mice, at the phylum level Firmicutes was increased and Bacteroidetes was decreased compared to the HFD-sham mice (Fig. 4A). Among the HFD + VDD-VSG mice, Firmicutes was increased and Bacteroidetes was decreased compared to the HFD-VSG mice.

The symbiotic bacterium Akkermansia muciniphila, which accounted for 3% of fecal bacteria in the HFD-sham mice, was increased to 7% in the HFD-VSG mice. In the HFD + VDD-sham group, the ratio of Akkermansia muciniphila decreased to 2%, whereas it was increased to 5% in the HFD + VDD-VSG group. We also applied 16S rDNA high-throughput sequencing to examine the gut microbiota of the mice. Through hierarchical clustering (Fig. 4B,C), we observed increased phylum Firmicutes and decreased Bacteroidetes phylum of the VSG mice compared to the sham-operated mice. Again, the increased phylum Firmicutes and decreased Bacteroidetes phylum of the HFD + VDD-VSG mice were suppressed compared to the HFD-VSG mice. The loss of Bacteroidetes and Akkermansia by the obese mice was restored after VSG (Fig. 4D,E). The results shown that a normal level of vitamin D could alleviate gut dysbiosis in obese mice after VSG sugery.

## Discussion

Obesity is becoming increasingly prevalent worldwide, and obesity and its associated comorbidities have imposed great health challenges and an enormous economic burden on societies. Epidemiologic studies have shown that vitamin D deficiency is associated with obesity^27–29^. Obese people are more likely to have vitamin D deficiency, and an inverse relationship between body mass index (BMI) and serum 25(OH)D was found in obese humans. But the precise mechanisms is not clear. A report has demonstrated that adipose tissue plays an vital active role in the regulation of vitamin D metabolism observed during obesity^46^. The other study indicated that abnormal regulation of serum 1,25(OH)2D is associated with major vitamin D metabolizing enzymes 1a hydroxylase and 24-hydroxylase in obesity, which are modified under high-fat diet, thus contributing to the obesity-related reduction of free 25(OH)D^42^.

Obesity is also characterized as a chronic low-grade inflammatory disease^47,48^. Studies have shown that LPS is the key factors, and play a crucial in obesity, such as LPS translocation could be enhanced by high-fat meals probably through chylomicron^49–51^. In addition, many evidence indicated that rodent models showed that a high-fat diet induces microbiome modifications in order to increase the intestinal permeability, which lead to enhance LPS translocation^52–56^.

Bariatric surgery has been reported to result in important changes in the composition of the gut microbiota, including increases in Proteobacteria and decreases in Clostridium, Bacteroides, and Prevotella^57^. A clinical study^57^ described that after a sleeve gastrectomy in subjects with obesity, an increase in the abundance of Akkermansia muciniphila which has a significant ability to degrade mucin and prevent inflammation and adipose tissue alterations^58^. Bariatric surgery is also able to modify the gut microbiota and increase the bacterial diversity, which might influence the circulating levels of LPS^59^.

Previous study showed that baseline glucose was significantly higher in mice which were fed with high-fat diet for 1 week, and a stable hyperglycemia and insulin resistance appeared over time^42^. However, VSG surgery could provides an substantial improvement of glucose^43^. Our present findings showed that the metabolic benefits of a VSG including considerable weight loss, reduced visceral adipose tissue, and improved glucose tolerance and insulin sensitivity are consistent with the results of clinical trials and other rodent studies^31–33,60,61^. In addition, the body weights of the HFD mice and the HFD + VDD mice were significantly different at 9 weeks after VSG, and compared to the HFD-VSG mice, the HFD + VDD-VSG mice had more visceral adipose tissue and higher transaminase and plasma LPS levels. Our analysis of the TJ proteins related to intestinal permeability revealed that the mRNA expressions of occludin, ZO-1, and claudin-2 of the ileum of the HFD + VDD-VSG mice were lower than those of the HFD-VSG mice.

Airu Zhu et al. indicated that vitamin D deficiency aggravated the impact of high-fat diet, and lead to robust metabolic disorders^62^. And Su et al. demonstrated that vitamin D could maintains gut eubiosis to antagonize Metabolic syndrome through induction of Paneth cell specific α-defensins^8^. Futhermore, some studies showed the mechanism that vitamin D could improve metabolic syndrome in high-fat diet is up regulation of tight junction components and antimicrobial peptides, partly^63^. Therefore, in our study, we detected the function of normal level of vitamin D in metabolic disorders of VDD in post-VSG obese mice.

SCFAs are produced from non-digestible polysaccharides by gut microbes^64^. SCFAs can also alter the composition of the microbiota^65,66^. The most well-known SCFAs are acetate acid, butyrate acid, propionate acid, and lactate acid. It is well-known that the increased SCFA levels are important for weight regulation because SCFAs can cause satiety so as to decrease the food intake^51^. Further, SCFAs may play important roles in body weight regulation via increasing the total energy expenditure^67^. Also, SCFAs facilitate the absorption of minerals via adjusting the intestinal pH. Meanwhile, SCFAs serve as a substrate of colonocytes (particularly butyrate) to induce a increased portion of beneficial bacteria^68–70^.

We analyzed the pH and the changes in the SCFAs of the cecal contents of mice that had undergone a VSG, and the results showed increased contents of acetic acid and butyric acid in the HFD-VSG mice and HFD + VDD-VSG mice, whereas the pH values were decreased in both groups, which is consistent with another report^71^. In our comparison of the HFD-VSG mice and HFD + VDD-VSG mice, we observed that the contents of acetic acid and butyric acid in the feces of the HFD + VDD-VSG mice were decreased, and the pH was increased. Our analysis of the changes in fecal microbes revealed that the abundance of Firmicutes was reduced whereas the abundance of Bacteroidetes was greatly increased in the HFD-VSG mice and HFD + VDD-VSG mice; the abundance of Akkermansia muciniphila was also increased in these mice. However, we also analyzed the changes in fecal microbes between the HFD-VSG and HFD + VDD-VSG mice. The increase in the abundance of Bacteroidetes and Akkermansia muciniphila in the HFD + VDD-VSG mice was less than that in the HFD-VSG mice, and the reduction in the abundance of Firmicutes in the HFD + VDD-VSG mice was smaller than that in the HFD-VSG mice.

Taken together, our findings demonstrated that a deficiency in vitamin D can weaken the effects of VSG including maintaining weight loss, insulin resistance, intestinal inflammation and permeability improvement, and a restoration of the gut dysbiosis. We thus propose that a normal circulating concentration of vitamin D is important for the improvement of metabolic status after VSG.

## Supplementary Information

Supplementary Information.

## Data Availability

All experimental data used to support this findings are included within the article.
