# Supplementary material for: Effects of vitamin D deficiency on the improvement of metabolic disorders in obese mice after vertical sleeve gastrectomy
Source: Sci Rep. 2021 Mar 16;11:6036. doi: 10.1038/s41598-021-85531-9 (PMC7971024; doi:10.1038/s41598-021-85531-9)
Supplement: Supplementary file 1 — Supplementary Information. [file 41598_2021_85531_MOESM1_ESM.docx]

**Effects of vitamin D deficiency on the improvement of metabolic disorders in obese mice after vertical sleeve gastrectomy**

Jie Zhang^1,2#^, Min Feng^3#^, Lisha Pan^4#^, Feng Wang^1,3^, Pengfei Wu^4^, Yang You^4^, Meiyun Hua^4^, Tianci Zhang^4^, Zheng Wang^5^, Liang Zong^6^*, Yuanping Han^4^* and Wenxian Guan^1,3,^*

**Supplementary figure**

**
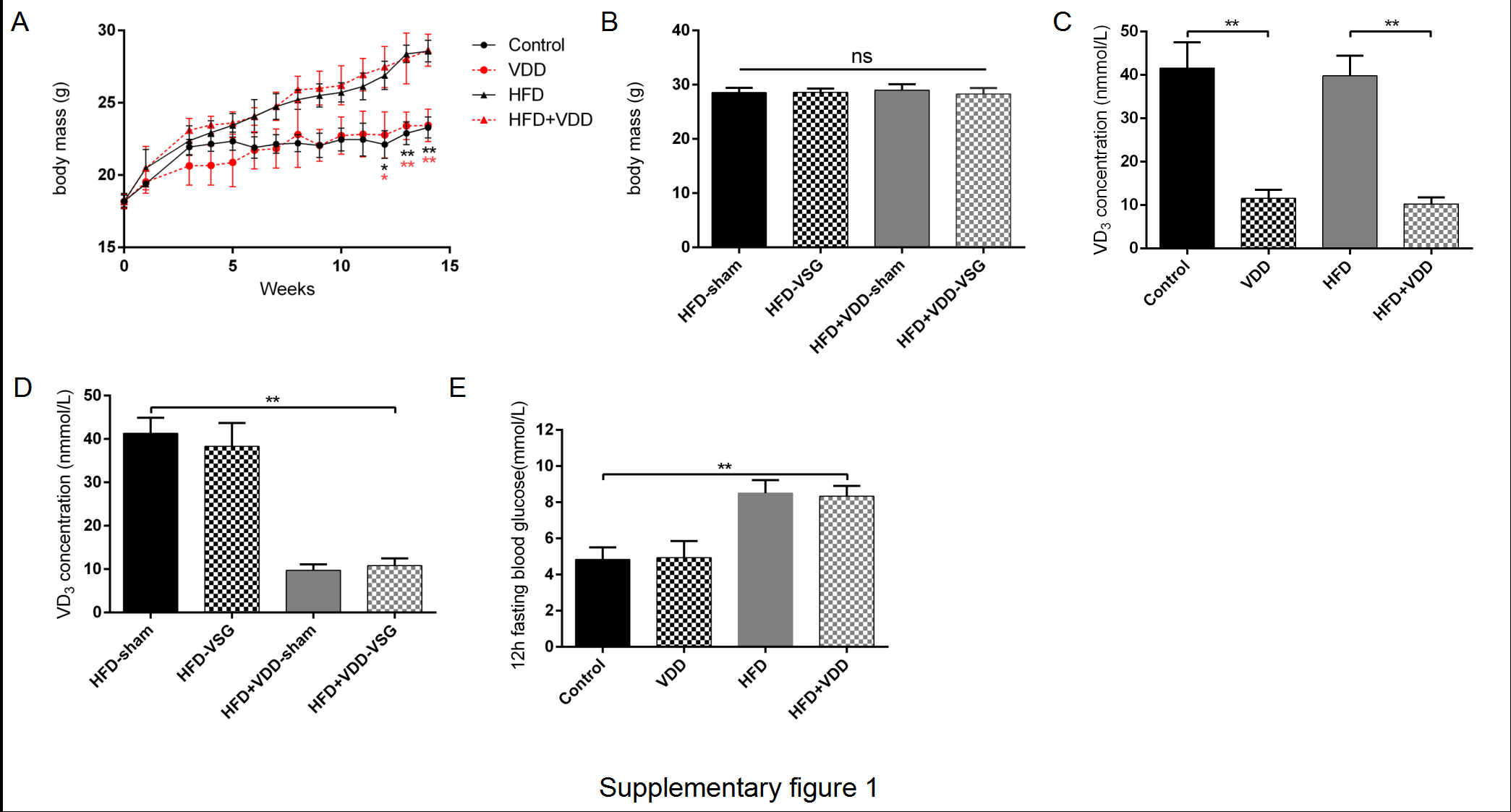
**

**Figure S1**: A and B: The body weights of the DIO and normal diet mice. C and D: The plasma VD3 concentrations of the HFD+VDD and the HFD mice. E: The blood glucose levels of the DIO mice.


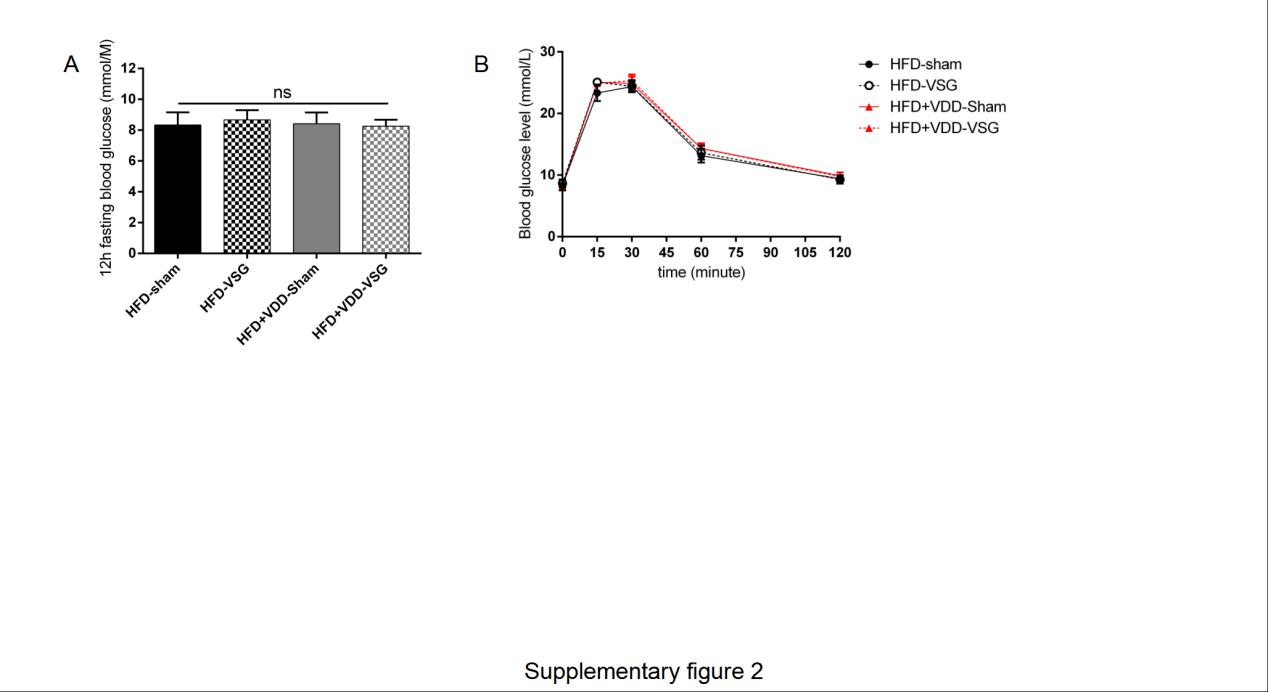


**Figure S2**: A: 12h fasting blood glucose levels before VSG surgery. B: Blood glucose levels before VSG surgery.
